# Botanical drugs for treating erectile dysfunction: clinical evidence

**DOI:** 10.3389/fphar.2023.1232774

**Published:** 2023-08-16

**Authors:** Dengjianyi Xu, Yucong Zhang, Jian Bai, Huixing Yuan, Tao Wang, Jihong Liu, Wen Song, Delin Ma

**Affiliations:** ^1^ Department of Urology, Tongji Hospital, Tongji Medical College, Huazhong University of Science and Technology, Wuhan, Hubei, China; ^2^ Department of Geriatrics, Tongji Hospital, Tongji Medical College, Institute of Gerontology, Huazhong University of Science and Technology, Wuhan, China; ^3^ Reproductive Medicine Center, Tongji Hospital, Tongji Medical College, Huazhong University of Science and Technology, Wuhan, Hubei, China; ^4^ Department of Endocrinology, Tongji Hospital, Tongji Medical College, Huazhong University of Science and Technology, Wuhan, China

**Keywords:** botanical drugs, natural products, erectile dysfunction, clinical study, phosphodiesterase-5

## Abstract

Phosphodiesterase-5 inhibitors (PDE5-i) have been widely used in clinical practice for the treatment of erectile dysfunction (ED). However, due to its suboptimal therapeutic effects and side effects, it is necessary to develop new medicines for ED treatment. Botanical drugs have been widely investigated as potential ED treatment drugs and have shown promising therapeutic effects. This review summarized 34 studies, including five botanical drugs with PDE5 inhibitory activity, seven botanical drugs without PDE5 inhibitory activity, and six mixed botanical drugs. The results of clinical studies regarding the aforementioned botanical drugs and relevant mechanisms are summarized in this study. It is necessary to conduct high-quality clinical trials to verify the dosage, targeted patients and therapeutic effects, and further pharmacology experiments are also needed to identify the active compounds.

## 1 Introduction

Erectile dysfunction (ED) is the persistent or recurrent inability to achieve or maintain an sufficient erection for satisfying sexual intercourse requirements (Sin et al., 2021). Approximately 52% of non-institutionalized men aged 40–70 years were reported to suffer from some degree of ED, and the prevalence of ED is predicted to reach 322 million cases by 2025 (Sin et al., 2021).

The physiological basis of penile erection is the dilation of the penile artery and the relaxation of the trabecular corpus cavernosum. When the penile artery and the smooth muscle in the trabecular region contract, the penis is in a relaxed state. In contrast, when the penile artery and the smooth muscle in the trabecular bone relax, the penis is in an erection state. The nitric oxide/cyclic guanosine monophosphate (NO/cGMP) signalling pathway plays an important role in the physiological basis of penile erection. After receiving sexual stimulation, NO is released from neurons and vascular endothelial cells in the penis sponge. Then, NO activates guanylate cyclase (GC) in the smooth muscle cells of the cavernous body. GC can catalyse the transformation of guanosine triphosphate (GTP) into cGMP. cGMP can activate protein kinase G (PKG), which will reduce the flow of Ca^2+^ into smooth muscle cells. When Ca^2+^ in the smooth muscle cells decreases, the smooth muscle cells of the cavernous body relax, and blood flows into the cavernous sinus increase. This process will finally cause the erection of the penis. In this process, PDE5 can breakdown cGMP into inactive guanosine-5′-monophosphate (5′-GMP), resulting in an increase in the flow of Ca^2+^ into smooth muscle cells, thus inhibiting penile erection (Anand Ganapathy et al., 2021).

Currently, the mainstream pharmacological treatment for ED is phosphodiesterase-5 inhibitors (PDE5-i). By inhibiting PDE5, the breakdown of cGMP is suppressed, which promotes penile erection. At present, the commonly used PDE5-i in clinical practice are avanafil, sildenafil, tadalafil, and vardenafil. However, the therapeutic effects of PDE5-i are not satisfactory. It has been reported that PDE5-i showed efficacy in only approximately 70% of patients, which is significantly lower in harder-to-treat subpopulations (Anand Ganapathy et al., 2021). Meanwhile, the adverse events of PDE5-i should also be noted. As the NO/cGMP signalling pathway exists in many different tissues and cells, such as the urinary system, respiratory system, digestive system, visual system, central nervous system and immune system (Anand Ganapathy et al., 2021), PDE5-i can also cause side effects in these systems. Common adverse events of PDE5-i include flushing and headaches (Sudyka and Wick, 2021), while more serious side effects may include non-arteritic anterior ischemic optic neuropathy (NAION), hearing loss, nasopharyngitis and priapism (Anand Ganapathy et al., 2021).

Overall, it is necessary to develop new drugs for the treatment of ED, and botanical drugs can provide important sources. Many botanical drugs have been used as medicines to treat diseases for centuries. Among them, botanical drugs are used as “aphrodisiacs” when treating ED. Even today, many plants are still used as “aphrodisiacs” to enhance libido, treat ED, and other purposes. This article summarizes the clinical research results and mechanisms of 12 single botanical drugs and 6 mixed botanical drugs.

In this review, botanical drugs were categorized based on whether they were found to have PDE5 inhibitory activity (Tables 1, 2). The possible mechanisms of the non-PDE5 inhibitor botanical drugs were also discussed.

**TABLE 1:** Botanical drugs with phosphodiesterase-5 inhibitory activity.

Botanical drugs	Type of study	Study subject	Intervention measures	Therapeutic effects	Adverse events	Mechanism of action Sin et al. (2021)
*Eurycoma longifolia*	Randomized double-blind placebo	45∼49 years men with ADAM and serum total testosterone levels ≤346 ng/dL	Two capsules (each containing 200 mg of standardized *E. longifolia* dry extract) per day and 60-min concurrent-training session three times weekly for 6 months	Erectile function improved and total testosterone level increased. Jansakul et al. (2012)	Not mentioned	1) Interfere with Ca^2+^ mobilization to relax smooth muscle tension of corpus cavernosum 2) Antagonize angiotensin II-induced contraction via inhibition of angiotensin II type l receptor and enhance bradykinin-induced relaxation through inhibition of ACE 3) Inhibition of PDE5
	Randomized double-blind placebo	30∼55 years healthy men	Two capsules of *E. longifolia* water extract 2 times a day after lunch and dinner for 12 weeks	Physical Functioning domain of SF-36 improved. Overall erectile function domain in IIEF increased. Significant improvement in fat mass lost in subjects with BMI ≥ 25 kg/m^2^. Chaturapanich et al. (2008)	1) URTIS	
					2) Generalised body ache	
					3) Conjunctivitis	
					4) Infected chalazion	
					5) Herpes zoster	
					6) Ankle pain	
					7) Archilles tendinitis	
					8) R index finger pain	
					(accessed as “unlikely”)	
*Tribulus terrestris*	Randomized double-blind placebo	18∼65 years patients with mild or moder-ate ED and with or without hypoactive sexual desire disorder	3 tablets (each contains 250 mg *T. terrestris* extracts) per day after meals for 12 weeks	GEQ and IIEF scores improved. Khanavi et al. (2012)	Abdominal pain	1) Regulate the NO/NOS pathway and corpus cavernosum endothelium 2) activate the Nrf2/HO-1 pathway, inhibit NF-κB levels, increase serum testosterone levels 3) increase serum DHEAS levels 4) PDE5 inhibition
	Before-after study	40∼70 years patients with PADAM, complaining mainly of ED and low libido	Three times daily (250 mg extracts per dose) for 3 months	Total and free testosterone and IIEF-5 improved versus baseline	Not mentioned	
				IIEF-5 had correlation with total testosterone, free testosterone. Gauthaman and Ganesan, (2008)		
	Randomized single-blind placebo	40∼70 years patients suffering from ED and partial androgen deficiency	Three times daily for 3 months, each capsule containing 250 mg of *T. terrestris*	IIEF-5 and total testosterone level improved. PSA-t increased. Sahin et al. (2016)	Not mentioned	
	Randomized double-blind placebo	Healthy men with self-reported ED	400 mg *T. terrestris* twice daily for 30 days	No significant improvement in the IIEF-5 score and serum testosterone. Do et al. (2013)	Not mentioned	
*Butea superba*	Open label study (sildenafil as control group)	42∼78 years patients with organic ED	Two capsules (50 mg *B. superba* per capsule) 1 or 2 h before each sexual encounter	The results could not be repeated, and there were no differences between the groups. Chiou and Wu, (2012)	Headache and flushing (one patient)	PDE5 inhibition
	Randomized double-blind placebo	30∼70 years patients with ED	Two capsules per day (250 mg dried *B. superba* tubers per capsule)for first 4 days and then 4 capsules per day for total of 3 months	4 domains in IIEF-5 improved. No change in testosterone level. Tee et al. (2017)	Not mentioned	
*Ginkgo biloba*	Randomized double-blind placebo	36∼60 years patients (no limit on gender) taking any anti-depressant drug and experiencing sexual problems	120 mg *G. biloba* daily for the first 2 weeks, 160 mg for the second 2 weeks and 240 mg for the last 4 weeks	There were no statistically significant differences versus the placebo. Oboh et al. (2018)	1) Gastrointestinal disturbances	PDE5 inhibition
					2) Sedation	
					3) Headache	
					4) Increased oral intake	
	Randomized triple-blind placebo	23∼66 years patients (no limit on gender) taking any anti-depressant drug for at least 2 weeks and experiencing sexual problems	125 mg *G. biloba* daily in the first week and then increased to 240 mg daily for the last 11 weeks. The dosage decreased to 120 mg only when gastric irritation occurred	There were no statistically significant differences versus the placebo. No differences in side-effects. Li et al. (2019)	1) Had to omit	
					2) Gastric pain	
					3) Nausea	
					4) “Muzzy head”	
					5) Anaesthesia or paraesthesia fingers	
*Kaempferia parviflora*	Open-label study	50∼68 years males with self-reported mild ED	One capsule containing 100 mg of KaempMax™ (a *K. parviflora* rhizome extract standardized to 5% DMF) daily for 1 month	IIEF total score, domains of erectile function and intercourse satisfaction improved. Modabbernia et al. (2012)	None	1) Increase blood flow in the testis
						2) Act as an L-type Ca^2+^ channel inhibitor, inhibit the intracellular mobilization of Ca^2+^
						3) PDE5 inhibition

PDE5, phosphodiesterase-5; ADAM, androgen deficiency of aging males; SF-36, Quality of Life questionnaire; IIEF, international index for erectile function; BMI, body mass index; URTIS, upper respiratory tract infections; ACE, angiotensin converting enzyme; ED, erectile dysfunction; GEQ, global efficacy question; PADAM, partial androgen deficiency; PSA-t, total prostate-specific antigen; NO, nitric oxide; NOS, nitric oxide synthase; Nrf2, nuclear factor-erythroid 2-related factor 2; HO-1, heme oxygenase-1; NF-κB, nuclear factor kappa-B; DHEAS, dehydroepiandrosterone sulfate; DMF, 5,7-dimethoxyflavone.

**TABLE 2:** Botanical drugs without phosphodiesterase-5 inhibitory activity.

Botanical drugs	Type of study	Study subject	Intervention measure	Therapeutic effects	Adverse events	Mechanism of action Sin et al. (2021)
*Lepidium meyenii* (Maca)	Double-blind randomized parallel placebo	>40 years men with AMS score ≥27	1,000 mg of Maca, two pills at a time, three times per day for 12 weeks before food intake	AMS, IIEF, ADAM, total and free testosterone levels improved. Al-Rehaily et al. (2015)	Gastrointestinal disorders	Nil
	Double-blind randomized placebo	31∼41 years patients with mild ED	1,200 mg Maca dry extract two times daily for 12 weeks	IIEF-5 score improved. Psychological, physical and social performance-related SAT-P improved. Kim et al. (1998)	None	
	Double-blind randomized placebo parallel	21∼56 years healthy men	3 tablets of 500 mg each of gelatinized Maca per day (Maca 1.5 g) for 12 weeks, or 6 daily tablets of gelatinized Maca (3,000 mg) for 12 weeks	Sexual desire improved, and not because of changes in mood or serum testosterone and oestradiol levels. Lin and Gou, (2013)	Not mentioned	
	Double-blind randomized placebo cross-over	Experienced and endurance trained male cyclists, mean age 30 ± 7 years old	5 capsules per day (each containing 400 mg *L. meyenii* root’s extract) for 2 weeks and 1 week washout period	Self-rated sexual desire score improved. Li et al. (2014)	Not mentioned	
*Rosa damascena*	Double-blind randomized placebo	18∼45 years patients with opioid-dependency and treated with metha-done maintenance therapy and have sexual dysfunction as side-effect	2 mL/day and contained 17 mg citronellol of essential oil of *R. damascena* for 8 weeks	Sexual dysfunction decreased, and serum testosterone increased. No consistent relation between them. Chen and Lee, (1995)	Not mentioned	Nil
	Double-blind randomized placebo	Patients treated with an SSRI and suffering from major depressive disorders and SSRI-I SD	2 mL of verum daily (17 mg citronellol of essential oil of *R. damascena*) for 8 weeks	Sexual dysfunction improved, and depression symptoms reduced as sexual dysfunction improved. Wang et al. (2010)	None	
*Crocus sativus*	Double-blind randomized placebo	18∼45 years male patients with major depressive disorder stabilized on fluoxetine and fluoxetine-related sexual dysfunction	Saffron capsule 15 mg twice per day for 4 weeks	Erectile function, intercourse satisfaction, and IIEF total scores improved	1) Nausea	Increase in cGMP levels
					2) Daytime drowsiness	
					3) Decreased appetite	
					4) Dry mouth	
					5) Nervousness	
					6) Restlessness	
					7) Morning drowsiness	
					8) Increased appetite	
	Double-blind randomized placebo parallel	40∼76 years patients with ED and diabet	Rub a pea-sized amount of the gel containing 1% *C. sativus* on penis half an hour before a sexual intercourse	Erectile dysfunction improved. Ying et al. (2018)	None	
	Open-label study	26∼62 years patients with ED	1 tablet (200 mg *C. sativus* per tablet)every morning for 9 days, 2 tablets on day 10	IIEF-15 improved. NPT test improved. Cho et al. (2013)	Not mentioned	
	Randomized open-label cross-over	27∼60 years patients with ED	Saffron (30 mg, the extract of *C. sativus*) twice daily for 12 weeks, 2-week washout phase and then on-demand 50-mg sildenafil	No significant improvements in the IIEF sexual function domains, SEP questions and EDITS scores. Leitao et al. (2021)	1) Headache	
					2) Flushing	
					3) Dyspepsia	
					(Less than sidenafil)	
*Panax ginseng*	Double-blind randomized placebo parallel multicenter	Patients with mild-to-moderate ED	4 tablets of SKGB (350 mg ginseng berry extract per tablet), daily, for 8 weeks	The total and each of the individual domain scores of IIEF-15 increased. PEDT scores improved. Ismail et al. (2012)	None	1) Regulate L-arginine/NO pathway, act as an NO donor to cause relaxation 2) regulate the NO/cGMP pathway, restore the corpus cavernosum endothelial function 3) inhibit oxidative stress, restore Akt activity, protect endothelial and smooth muscle cells (diabetes-induced ED) 4) enhance NO release from corpus cavernosum endothelial and nerve cell 5) increase serum testosterone levels 6) activate large conductance Ca2+-activate K+ (BKCa) channels in corporal smooth muscle cells 7) decrease HIF-1α expression in hypoxia state 8) increase eNOS expression 9) inhibit PDE5A
	Double-blind randomized placebo	33∼79 years patients with ED	1,000 mg of TMGE twice a day for 8 weeks	IIEF-15 total score and every individual domain of IIEF-5 improved. Kamenov et al. (2017)	Minor headaches	
	Double-blind randomized placebo	26∼70 years patients with mild or mild to moderate ED	1,000 mg Korean Red Ginseng (KRG) 3 times daily for 12 weeks	IIEF-5 total score, and scores on rigidity, penetration, maintenance improved. Roaiah et al. (2016)	Headache Insomnia	
*Trigonella foenum‐graecum*	Double-blind randomized placebo	25∼52 years healthy men	2 tablets per day (600 mg Testofen, a standardized *T. foenum‐graecum* extract, per day)for 6 weeks	DISF‐SR total scores and all four DISF-SR domains improved. GamalEl Din et al. (2019)	Slight stomach discomfort without food intake	Increase testosterone, LH, and FSH levels and decrease GnRH levels
	Double-blind randomized placebo	43∼70 years healthy men	*T. foenum-graecum* seed extract (Testofen) at a dose of 600 mg/day for 12 weeks	AMS score improved	Dizziness headache	
				Sexual function, including number of morning erections and frequency of sexual activity improved		
				Both total and free serum testosterone increased. Santos et al. (2014)		
*Pistacia vera*	Prospective study	38∼59 years patients with ED for at least 12 months	100 g pistachio nuts at lunch diet for 3 weeks	Mean peak systolic velocity values, IIEF-15 total scores and all five IIEF-5 domains improved	None	Nil
				TC and LDL levels decreased, HDL level increased. Cortes-Gonzalez et al. (2010)		
*Withania somnifera* (Ashwagandha)	Single-blind randomized placebo parallel	18∼60 years patients with psychogenic ED and serum testosterone levels over 239 ng/dL	4 Ashwagandha tablets (500 mg each) thrice a day after food for 60 days	Both Ashwagandha and placebo provided no relief in psychogenic ED. Cherdshewasart and Nimsakul, (1008)	Not mentioned	Activating the Nrf2/HO-1 pathway, inhibit NF-κB levels, and increase serum testosterone levels

PDE5, phosphodiesterase-5; AMS, Aging Males’ Symptoms scale; IIEF, international index for erectile function; ADAM, androgen deficiency of aging males; ED, erectile dysfunction; SAT-P, satisfaction profile; SSRI, selective serotonin reuptake inhibitor; SSRI-I SD, selective serotonin reuptake inhibitor induced sexual dysfunction; NPT, nocturnal penile tumescence; SEP, sexual encounter profile; EDITS, erectile dysfunction inventory of treatment satisfaction; cGMP, cyclic guanosine monophosphate; SKGB, standardized Korean ginseng berry; PEDT, premature ejaculation diagnostic tool; TMGE, tissue-cultured mountain ginseng extract; NO, nitric oxide; HIF-1α, hypoxia inducible factor-1α; eNOS, endothelial nitric oxide synthase; DISF‐SR, Derogatis Interview for Sexual Functioning-Self Report; LH, luteinizing hormone; FSH, follicle-stimulating hormone; GnRH, gonadotropin releasing hormone; TC, total cholesterol; LDL, low density lipoprotein; HDL, high density lipoprotein; Nrf2, nuclear factor-erythroid 2-related factor 2; HO-1, heme oxygenase-1; NF-κB, nuclear factor kappa-B.

## 2 Mechanism of botanical drugs regarding ED treatment

Among 12 single botanical drugs, 5 plants show PDE5 inhibitor activity, while 7 do not (Anand Ganapathy et al., 2021; Sin et al., 2021). Apart from PDE5 inhibitory activity, the potential anti-ED mechanisms of the botanical drugs are as follows: 1) regulating NO-related pathways, 2) activating the nuclear factor-erythroid 2-related factor 2/heme oxygenase-1 (Nrf2/HO-1) signaling pathway and inhibiting nuclear factor kappa-B (NF-κB), and 3) increasing testosterone levels. As mentioned above, through the NO/cGMP pathway, increasing NO can enhance cavernosal smooth muscle relaxation and penile erection. NO is synthesized from L-arginine. NO synthase isoforms, neuronal nitric oxide synthase, endothelial nitric oxide synthase (eNOS), and inducible NOS are the main enzymes responsible for the synthesis of NO. The Nrf2/HO-1 signalling pathway plays a key role in the oxidative stress response, and Nrf2 is a significant transcription factor that promotes the expression of many antioxidant genes. HO-1 is an inducible isoform protein and can be induced by oxidative stress and many other conditions. NF-κB is an enhancer-binding transcription factor and participates in the immune response, cell proliferation and apoptosis. By activating the Nrf2/HO-1 pathway and inhibiting NF-κB, oxidative stress, an important factor in the development of ED, can be inhibited. Testosterone also plays a significant role in penile erection function in central and peripheral ways. Thus, increasing testosterone has positive effects on erectile function.

### 2.1 Mechanisms of botanical drugs with PDE5 inhibitory activity

Among the plants with PDE5 inhibitory activity listed in Table 1, *Kaempferia parviflora* Wall. ex Baker (Zingiberaceae), *Tribulus terrestris* L. (Zygophyllaceae), and *Eurycoma longifolia* Jack (Simaroubaceae) show activities other than PDE5 inhibitors (Anand Ganapathy et al., 2021; Sin et al., 2021).

*K. parviflora* has been used in Thai traditional medicine to treat various diseases. It is believed by the locals that it could improve male erectile function. However, the subjective experience by users is not sufficient, and some studies have been conducted. There are indeed some active ingredients in *K. parviflora* that may improve erectile function. 3,5,7,3’,4’-Pentamethoxyflavone, an active ingredient of *K. parviflora*, can act as an L-type Ca^2+^ channel inhibitor to reduce intracellular Ca^2+^ to induce relaxation of the isolated human cavernosum (Jansakul et al., 2012). In Sprague‒Dawley rats (S-D rats), a 95% v/v ethanol extract of *K. parviflora* containing phenols as its major component, and flavonoids as its minor component increased blood flow in the testis (Chaturapanich et al., 2008).

*T. terrestris* has been recognized as a botanical drug with anti-inflammatory, antidiabetic and anticancer functions. *T. terrestris* has multiple effects that contribute to erectile function. A study used human platelets as a rich source of PDE enzymes, in which an 80% v/v methanol extract of *T. terrestris* showed cGMP PDE inhibitory activity (Khanavi et al., 2012). In addition, protodioscin in *T. terrestris* increased serum testosterone, dehydroepiandrosterone sulfate (DHEAS) and dihydrotestosterone (DHT) levels in baboons, rhesus monkeys, New Zealand white rabbits, and S-D rats. These androgens are crucial not only for the development of male external genitalia and male secondary sexual characteristics, but also for sexual desire and nocturnal penile erections (Gauthaman and Ganesan, 2008). Moreover, a study found that 70% v/v methanol extract of *T. terrestris* activated the Nrf2/HO-1 pathway, inhibited NF-κB in reproductive tissues, and increased serum testosterone levels in S-D rats. In this study, researchers showed that *T. terrestris* can inhibit the development of ED in an antioxidant way (Sahin et al., 2016). Similarly, in the corpus cavernosum of New Zealand white male rabbits, a 90% v/v methanol extract of *T. terrestris* regulated the NO/NOS pathway and relaxed the corpus cavernosum endothelium. By activating the NO/NOS pathway in the corpus cavernosum endothelium, NO was increased, and the NO/cGMP pathway was activated (Do et al., 2013).

*E. longifolia*, also named Tongkat Ali by locals in Malaysia, is a well-known botanical drug used as an aphrodisiac. Animal studies found that *E. longifolia* was able to improve libido and erectile function (Chiou and Wu, 2012). A β-carboline alkaloid, isolated from *E. longifolia*, 9-hydroxycanthin-6-one, was found to interfere with Ca^2+^ mobilization to relax the smooth muscle tension of the corpus cavernosum and seminal vesicle in the corpus cavernosum of S-D rats (Chiou and Wu, 2012). In addition, a dichloromethane (DCM) subfraction of 95% v/v ethanol tincture extract of *E. longifolia* antagonized angiotensin II (Ang II) induced contraction via inhibition of Ang II type l receptor and enhanced bradykinin (BK)-induced relaxation through inhibition of acetylcholinesterase (ACE) in rat corpus cavernosum. BK can induce corpus cavernosum relaxation, and Ang II can induce corpus cavernosum contraction. The renin-angiotensin system in the corpus cavernosum is important for maintaining corpus cavernosal tone (Tee et al., 2017). Moreover, some phenolic-rich extracts from *E. longifolia*, containing ellagic acid, rutin and quercetin, were reported to inhibit PDE5, arginase and ACE in penile tissues of Wistar albino rats (Oboh et al., 2018).

### 2.2 Mechanisms of botanical drugs without PDE5 inhibitory activity

Four plants that may promote erectile function, including *Withania somnifera* (L.) Dunal (Solanaceae), *Trigonella foenum-graecum* L. (Fabaceae), *Crocus sativus* L. (Iridaceae) and *Panax ginseng* C.A.Mey. (Araliaceae) (Sin et al., 2021) (Table 2).

*W. somnifera* is an important botanical drug in traditional Indian medicine. It is reported to have anticancer, antioxidant, antistress, and immunomodulatory functions. In one study, 70% v/v methanol extract of *W. somnifera* was found to activate the Nrf2/HO-1 pathway, inhibit the NF-κB level in reproductive tissues of S-D rats, and increase serum testosterone levels in S-D rats (Sahin et al., 2016).

Seeds of *T. foenum-graecum* contain high level polyphenolic flavonoids, which possess reproductive protective effects and antioxidant, antidiabetic and anti-inflammatory functions. Vitexin, an active flavonoid of *T. foenum-graecum*, may modulate the hypothalamus-pituitary-gonadal axis to decrease serum gonadotropin releasing hormone (GnRH) levels, increase serum luteinizing hormone (LH) and follicle-stimulating hormone (FSH) levels, and increase serum testosterone levels in streptozotocin-induced diabetic mice. Therefore, vitexin may regulate androgen generation, enhance sex performance, and improve diabetic-induced fertility impairments (Li et al., 2019).

*C. sativus* is widely used in Iran, India, Greece, Spain, and Italy. It is believed by locals that it has an aphrodisiac effect (Modabbernia et al., 2012). In one study, the hexane fraction from the alcoholic extract of *C. sativus* increased cGMP levels in Wistar rats. This indicates that *C. sativus* might have active ingredients that can improve erectile function by increasing cGMP levels in corporal smooth muscle cells (Al-Rehaily et al., 2015).

*P. ginseng* is one of the most famous and most important botanical drugs in traditional Chinese medicine. It is believed to have multiple benefits for keeping healthy and has been used for thousands of years in China. Saponins have been found to be the major active ingredients in *P. ginseng*, which is responsible for the therapeutic effects. Multiple kinds of ginsenosides are considered to be the major active ingredients of saponins. A large number of studies have been performed to determine the mechanisms of *P. ginseng*, and some of them focused on reproductive effects. Saponins can regulate the L-arginine/NO pathway and act as NO donors to cause relaxation in the corpus cavernosum isolated from New Zealand White rabbits, regulate the NO/cGMP pathway and restore corpus cavernosum endothelial function in S-D rats, inhibit oxidative stress, restore Akt activity, and protect endothelial and smooth muscle cells in streptozotocin-induced diabetic rats (Kim et al., 1998; Lin and Gou, 2013; Li et al., 2014). Ginsenosides, the active ingredient complex of *P. ginseng*, enhanced NO release from corpus cavernosum endothelial and nerve cells in the corpus cavernosum of New Zealand white male rabbits *in vitro* (Chen and Lee, 1995). Ginsenoside Rg1 increased serum testosterone levels and regulated the NO/cGMP pathway in the corpus cavernosum of Kunming mice and New Zealand rabbits (Wang et al., 2010). Ginsenoside Rg3 activated large conductance Ca^2+^-activated K^+^ (BK_Ca_) channels in rat brain BK_Ca_ channels (Choi et al., 2011). Ginsenosides Rk1, Rk3, Rg5, and Rh4, the active ingredient complex of *P. ginseng*, inhibited PDE5A in ICR mice and primary corpus cavernosum smooth muscle cells. Rg5 also decreased hypoxia inducible factor-1α (HIF-1α) expression in hypoxic state and increased eNOS expression (Ying et al., 2018). *P. ginseng* berry extract GB0710, containing ginsenoside Re as a major component and ginsenosides Rb1, Rb2, Rc, Rd, Rg1, and Rg2 as minor components, regulated the NO/cGMP pathway to enhance relaxation in rabbit corpus cavernosum *in vitro* and S-D rats *in vivo* (Cho et al., 2013). In summary, by increasing NO and cGMP levels in the corpus cavernosum, *P. ginseng* relaxed corpus cavernosum smooth muscles and thus improved erectile function. *P. ginseng* also showed therapeutic effects on ED by its antioxidant and antistress functions.

Notably, the majority of studies did not solely study one single identified bioactive substance of plants. They studied crude plant extracts or semipurified fractions, both of which are mixtures of active substances. Some active substances may produce a synergistic action. Furthermore, some environmental factors, such as temperature, humidity and soil composition undergo alterations, may influence the presence and concentration of active substances within botanical drugs. These facts could in some way explain the following phenomenon: the pharmacological mechanisms for the same botanical drug varied from studies with different extraction methods. For example, three studies of *T. terrestris* used 70%, 80%, and 90% v/v methanol extracts. Researchers found that 70% methanol v/v extract activated the Nrf2/HO-1 pathway, inhibited NF-κB levels and increased serum testosterone levels, 80% v/v methanol extract inhibited PDE, and 90% v/v methanol extract regulated the NO/NOS pathway and corpus cavernosum endothelium. Two studies of *P. ginseng* used total ginsenosides and ginsenosides Rk1, Rk3, Rg5, and Rh4. Researchers found that total ginsenosides activated large conductance Ca^2+^-activated K^+^ (BK_Ca_) channels in corporal smooth muscle cells, while ginsenosides Rk1, Rk3, Rg5, and Rh4 inhibited PDE5A. Some studies analysed the pharmacological mechanisms of specific active substances, such as crocin in *C. sativus* and ginsenoside Rg1 in *P. ginseng*.

## 3 Clinical effects regarding botanical drugs with PDE5 inhibitory activity

### 3.1 *Eurycoma longifolia* Jack (Simaroubaceae)

The results of two clinical trials (randomized, double-blind, placebo-controlled) on *E. longifolia* significantly increased International Index of Erectile Function (IIEF) scores (Ismail et al., 2012; Leitao et al., 2021). Additionally, one of the studies found that *E. longifolia* increased serum testosterone levels, which was not reported in preclinical experiments. In this study, 45- to 49-year-old patients with androgen deficiency of aging males (ADAM) (total testosterone ≤ 346 ng/dL) were enrolled (Leitao et al., 2021). Moreover, another study also found that interventional subjects (30–55-year-old healthy men) with Body Mass Index (BMI) ≥ 25 kg/m^2^ showed significant improvement in fat mass loss after *E. longifolia* treatment (*p* = 0.008) (Ismail et al., 2012). The *E. longifolia* capsule in one clinical trial was produced by a local compounding pharmacy, containing 200 mg of standardized dry extract [2-dihydro-18-dedihydrolongilactone: 0.32% (w/w); 9-hydroxycanthin-6-one 0.49% (w/w); Eurycomanone: 1.21% (w/w); total protein: 26.3%; total polysaccharide: 28.8% ⋅ glycosaponin: 44.2%]. The duration of this study spanned a period of 6 months. Forty-five patients were recruited, and 8 dropped out (Leitao et al., 2021). The *E. longifolia* capsule in the other clinical trial contained 300 mg of a special freeze-dried water extract of *E. longifolia* root containing 75 mg of active ingredients. The duration of this study spanned a period of 3 months. A total of 109 patients were recruited (Ismail et al., 2012).

### 3.2 *Tribulus terrestris* L. (Zygophyllaceae)

There are four clinical trials on *T. terrestris*. Three of them found that *T. terrestris* significant improved ED (Roaiah et al., 2016; Kamenov et al., 2017; GamalEl Din et al., 2019), while one prospective, randomized, double-blind, placebo-controlled study did not, which may be because of the limited intervention period (Santos et al., 2014).

For 40–70-year-old patients with partial ADAM (PADAM) who mainly complained of ED and low libido, one before-after study found that *T. terrestris* (250 mg per dose, three times per day) increased both total and free serum testosterone levels (*p* = 0.026, *p* = 0.034, respectively), and there was a significant correlation between the increase in serum testosterone levels and the improvement in IIEF-5 scores (total testosterone: *p* = 0.001 and 0.054, before, *p* = 0.003 and 0.013, after; free testosterone: *p* = 0.001 and 0.025, before, *p* = 0.013 and 0.024, after). However, there were only 30 study subjects enrolled without control group. In this study, *T. terrestris* was collected from natural habitats during flowering. After air drying, rinsing with water, drying after evaporation of the solvent, powdering, extraction with 500 ml of 70% ethanol (or methanol or acetonitrile or hexane), and evaporation to dryness, the final dry extract was obtained. The duration of this study spanned a period of 3 months (Roaiah et al., 2016).

One randomized, double-blind, placebo-controlled study found that *T. terrestris* improved libido in patients with low sexual desire (intercourse satisfaction: *p* = 0.0005; orgasmic function, *p* = 0.0325; sexual desire: *p* = 0.0038; overall satisfaction: *p* = 0.0028). In this study, patients were required to orally intake Tribestan (Sopharma AD), an botanical medicinal product of Bulgarian origin, standardized with respect to furostanol saponins, calculated against protodioscin. Each Tribestan tablet contained 250 mg of the active substance *T. terrestris* herbal extractum siccum, and the content of furostanol saponins was no less than 112.5 mg. Moreover, it was reported that *T. terrestris* growing in different geographic regions of the world showed differences in the saponin content and the saponin composition, and *T. terrestris* used in this study was reported to have a high content of furostanol saponins of the diosgenin type. Patients aged 18–65 years with mild or moderate ED with or without hypoactive sexual desire disorder were enrolled. The duration of this study spanned a period of 3 months. A total of 180 patients were recruited, and 8 dropped out (Kamenov et al., 2017).

It is also worth noting that one randomized, single-blind, placebo-controlled study found that *T. terrestris* might slightly increase total Prostate Specific Antigen (PSA-t) levels (*p* = 0.007). In this study, 70 patients aged 40–70 years who suffered from ED and partial androgen deficiency (total testosterone < 3.5 ng/mL) were enrolled. They were required to take *T. terrestris* capsules (each containing 250 mg *T. terrestris* extracts) three times daily for 3 months. The *T. terrestris* extracts were standardized to contain no more than 45% steroidal saponins, but the detail process for the extraction was not mentioned in the study (GamalEl Din et al., 2019).

### 3.3 *Butea superba* Roxb. ex Willd. (Fabaceae)

In two studies on *B. superba*, the result of one open-label study showed that *B. superba* did not improve ED over the sildenafil group (Cortes-Gonzalez et al., 2010). It is worth noting that in this study, 100 mg of *B. superba* extract was given 1–2 h before each sexual encounter (Cortes-Gonzalez et al., 2010). However, in another study, *B. superba* was given with a larger dose regularly per day (Cherdshewasart and Nimsakul, 1008). In fact, in most studies, the experimental drug or placebo was given 1–3 times daily, rather than just before sexual intercourse. In addition, *B. superba* capsules in this study were obtained from BioC Ltd., Stockholm Sweden, a natural health product supplier. Researchers believed that PDE5-i was blended in the *B. superba* capsule. Furthermore, this study was not randomized, double-blind or placebo-controlled. Only 33 patients aged 42–78 years took *B. superba* capsules for only 1 week, and 1 patient dropped out. The period intervention and the sample size were limited (Cortes-Gonzalez et al., 2010).

Moreover, in the randomized, double-blind, placebo-controlled study, although *B. superba* did not increase serum testosterone levels, it did significantly improve ED symptoms and increase IIEF-5 scores, especially in domains 4 (almost never or never able to maintain erection after penetration) and 5 (difficult to maintain erection to completion of intercourse) (*p* < 0.05 and *p* < 0.01, respectively). In this study, *B. superba* was collected from Lampang Province. Their fresh tubers were cleaned, sliced into pieces, dried in a hot air oven, ground into fine powder, and passed through 100 mesh sieves. The powder was finally filled into capsules at 250 mg/capsule. 30–70 years old patients with ED were enrolled and required to orally take 2 capsules per day for the first 4 days and 4 capsules per day for the rest of the time. The duration of this study spanned a period of 3 months. 39 patients were recruited, and 8 dropped out (Cherdshewasart and Nimsakul, 1008).

It should be noted that the study subjects in both clinical trials were limited. In one study, 33 study subjects were included (Cortes-Gonzalez et al., 2010), while 39 study subjects were included and 8 dropped out in another study (Cherdshewasart and Nimsakul, 1008).

### 3.4 *Ginkgo biloba* L. (Ginkgoaceae)

Two clinical trials on *G. biloba* found that it did not improve ED symptoms (Kang et al., 2002; Wheatley, 2004). However, it is worth noting that both studies enrolled patients with sexual dysfunction caused by antidepressant drugs, especially selective serotonin reuptake inhibitors (SSRIs). Whether *G. biloba* has effect on other types of ED needs to be confirmed by more clinical trials.

In a randomized, triple-blind, placebo-controlled study, 24 patients aged 23–66 years were included, and 3 dropped out (Wheatley, 2004). In the randomized, double-blind, placebo-controlled study, thirty-seven patients aged 36–60 years were included, and 12 dropped out. Researchers believed that selection bias occurred in this study because some patients who subjectively experienced no sexual improvement did not participate in the second-month trial (Kang et al., 2002).

### 3.5 *Kaempferia parviflora* Wall. ex Baker (Zingiberaceae)

An open-label study on *K. parviflora* found that KaempMax™ (a *K. parviflora* rhizome extract standardized to 5% DMF) significantly improved the total score of IIEF (*p* = 0.0067), the score of erectile dysfunction domain (*p* = 0.0269), and the score of intercourse satisfaction domain (*p* = 0.0296) (Stein et al., 2018). Only 14 generally healthy males aged 50–68 years with self-reported mild ED were enrolled without control group (Stein et al., 2018).

## 4 Clinical effects regarding botanical drugs without PDE5 inhibitory activity

### 4.1 *Lepidium meyenii* Walp. (Brassicaceae)

Among the four randomized, double-blind placebo-controlled clinical trials regarding *L. meyenii* (Maca), two clinical trials found that *L. meyenii* significantly improved of IIEF (*p* < 0.001), International Prostatic Symptom Score (IPSS) (*p* = 0.001), Aging Males’ Symptoms scale (AMS) (*p* < 0.001), Androgen Deficiency in the Aging Males (ADAM) (*p* < 0.001), and Satisfaction Profile (SAT-P) (*p* < 0.05) (Zenico et al., 2009; Shin et al., 2023). In the other two studies, *L. meyenii* significantly increased the subjects’ sexual desire (*p* < 0.001, *p* = 0.03, respectively) (Gonzales et al., 2002; Stone et al., 2009). One study also found that the positive effect of *L. meyenii* on sexual desire was not because of changes in either Hamilton scores for depression or anxiety or serum testosterone and oestradiol levels. In addition, 1.5 g maca significantly improved sexual desire, similar to 3.0 g maca (Gonzales et al., 2002).

The detailed study designs of the 4 studies were summarized as follows: 1) men over 40 years of age with AMS total scores ≥27 were enrolled. The duration of this study spanned a period of 3 months. Eighty-eight patients were recruited, and 8 dropped out. 2 Maca extract capsules at a time, 3 times per day for 3 months (1,000 mg/capsule, containing 833 mg Maca gelatinized powder, n-benzyl-hexadecanamide between 115 and 175 μg/g, cadmium and total mercury less than 0.3 mg/kg, total arsenic and plumbum less than 0.5 mg/kg, absence of coliform bacteria) (Shin et al., 2023).

2) Patients aged 31–41 years with mild ED were enrolled. The duration of this study spanned a period of 3 months. Fifty patients were recruited. 1,200 mg Maca tablets 2 times daily for 3 months. The pulverized dehydrated Maca root tablets were directly imported from Peruvian Andes and kindly provided by Ibersan Srl, Forli, Italy (Zenico et al., 2009).
3) Twenty-one- to 56-year-old healthy men were enrolled. The duration of this study spanned a period of 3 months. Fifty-seven patients were recruited. Gelatinized maca tablet (500 mg maca per tablet, Laboratorios Hersil, Lima, Peru, provided), 3 tablets (1.5 g Maca) or 6 tablets (3.0 g Maca) per day for 3 months (Gonzales et al., 2002).
4) Experienced and endurance-trained male cyclists with a mean age of 30 ± 7 years were enrolled. As a crossover study, study subjects were required to take Maca or placebo for 2 weeks, with a 1-week washout period. Eight patients were recruited, and none dropped out. Maca root extract capsule (400 mg Maca per capsule, collected from the Cerro de Pasco region in the central Peruvian Andes Mountains, milled, soaked in 40°C water 3 times, filtered, solution concentrated under vacuum at 65°C, concentrated again to solid, mixed with modified starch and silica, spry dried to obtain a fine powder), 5 capsules (2.0 g Maca) per day for 2 weeks (Stone et al., 2009)

The number of sample sizes in the 4 studies was limited, and a pilot study only enrolled 8 healthy male cyclists, and Maca capsules were only taken for 2 weeks (Stone et al., 2009).

### 4.2 *Rosa damascena* Herrm. (Rosaceae)

There are two randomized, double-blind, placebo-controlled clinical trials on *R. damascena*. One study focused on 18–45-year-old patients with sexual dysfunction caused by methadone (Farnia et al., 2017), while the other focused on patients with sexual dysfunction caused by SSRIs (Farnia et al., 2015). Both studies used *R. damascena* oil 2 mL/day, containing 17 mg citronellol of essential oil of *R. damascena* (drops). The duration of both studies spanned a period of 2 months. One study recruited 50 patients with no drop-out (Farnia et al., 2017), and the other study recruited 68 patients with 8 drop-outs (Farnia et al., 2015). *R. damascena* was found to significantly improve sexual dysfunction caused by methadone and significantly increase serum testosterone levels, but there was no association between the two effects (Farnia et al., 2017). *R. damascena* also significantly improved both SSRI-induced sexual dysfunction and major depressive disorder (MDD) symptoms, and improve MDD symptoms occurred with the improvement in sexual dysfunction (Farnia et al., 2015). *R. damascena* might be used to treat sexual dysfunction caused by SSRIs.

There were also limitations in the two studies. The sample sizes were limited. Second, the inclusion criteria and single-center design could cause systematic selection bias. Third, the potential and unassessed physiological and psychological variables might also bring bias.

### 4.3 *Crocus sativus* L. (Iridaceae)

In four clinical trials regarding *C. sativus*, three studies found that *C. sativus* significantly improved the IIEF scores (*p* < 0.0001, *p* < 0.001, *p* < 0.001, respectively) (Shamsa et al., 2009; Modabbernia et al., 2012; Mohammadzadeh-Moghadam et al., 2015).

In addition, one study found that *C. sativus* significantly improved tip rigidity, tip tumescence, base rigidity, and base tumescence (*p* < 0.0001). This is an open-label study without placebo group. The intervention duration was only 10 days. In addition, only 20 patients aged 26–62 years were recruited. The optimum dose of *C. sativus was* not investigated (Shamsa et al., 2009).

One study found that *C. sativus* significantly improved the IIEF scores of ED patients with diabetes, and the intervention measure was to rub the *C. sativus* gel on patients’ penis half an hour before sexual intercourse, which is completely different from the daily timed and quantified oral administration used in most clinical trials. In this randomized, double-blind, placebo-controlled, parallel-group study, 40–76-year-old patients with ED and diabetes were enrolled and required to rub a pea-sized amount of the gel containing 1% *C. sativus* on their penis half an hour before sexual intercourse. Fifty patients were recruited, and none dropped out. The duration of the study spanned a period of 1 month (Mohammadzadeh-Moghadam et al., 2015).

One study found that *C. sativus* significantly improved the IIEF scores of patients with sexual dysfunction caused by fluoxetine, an SSRI. In this randomized, double-blind, placebo-controlled study, 18- to 45-year-old married male patients with MDD stabilized on fluoxetine and with fluoxetine-related sexual dysfunction were enrolled and required to take 15 mg *C. sativus* capsule twice per day. *C. sativus* was donated by Green Plants of Life Co., (IMPIRAN; Tehran, Iran). Each capsule contained 15 mg of dried *C. sativus* stigma extract and 1.65–1.75 mg of crocin, an important component of *C. sativus*. Thirty-six patients were recruited, and 6 dropped out. The duration of the study spanned a period of 1 month. In addition, because of the limited sample size, researchers failed to obtain significant differences in the orgasmic function domain (Modabbernia et al., 2012).

Moreover, one randomized, crossover, open-label clinical trial found that *C. sativus* did not improve the IIEF scores compared with sildenafil (Safarinejad et al., 2010). The duration of this study spanned 26 weeks. Patients were required to take 2 *C. sativus* capsules (each 15 mg, dried *C. sativus* stigma was produced by Novin Saffron Co., Mashhad, Iran) twice daily for 12 weeks followed by on-demand 50-mg sildenafil for another 12 weeks or *vice versa*, separated by a 2-week washout phase. A total of 346 patients were recruited, and 39 dropped out.

### 4.4 *Panax ginseng* C.A.Mey. (Araliaceae)

The results of three randomized, double-blind, placebo-controlled clinical trials regarding *P. ginseng* showed that *P. ginseng* significantly improved the IIEF scores (de Andrade et al., 2007; Kim et al., 2009; Choi et al., 2013).

One study found that *P. ginseng* significantly improved the domains of erectile function (*p* = 0.046), intercourse satisfaction (*p* = 0.005), orgasmic function (*p* = 0.002), sexual desire (*p* = 0.001), overall satisfaction (*p* = 0.001), and IIEF-15 total score (*p* = 0.002). In this study, researchers used 4-year-old *P. ginseng* berries to obtain standardized Korean ginseng berry (SKGB) extract. Patients were required to take 4 SKGB tablets (350 mg SKGB extract per tablet) per day. The duration of this study spanned a period of 2 months. A total of 119 patients were recruited, and 1 dropped out (Choi et al., 2013).

The other two studies showed similar results, with statistically significant improvements in all domains and IIEF-5 total score in the *P. ginseng* group but not in the placebo group (de Andrade et al., 2007; Kim et al., 2009). In addition, one study found that erectile function, intercourse satisfaction and overall satisfaction scores in the five domains of the IIEF were significantly higher in the *P. ginseng* group than in the placebo group (*p* < 0.05). The other study showed that scores on rigidity, penetration, and maintenance were significantly higher in the *P. ginseng* group than in the placebo group after treatment (*p* < 0.01) (de Andrade et al., 2007).

In one study, researchers used tissue-cultured mountain ginseng extract (TMGE). Patients were required to take 1,000 mg TMGE twice a day. Patients aged 33–79 years were enrolled. The duration of this study spanned a period of 2 months. A total of 143 patients were recruited, and 57 dropped out. Despite the staggering number of dropouts, 47 of them were in the placebo group because of no improvement in their ED. Considering the 68 patients contained in the placebo group at the beginning, only 21 patients in the placebo group finally finished the trial (Kim et al., 2009).

In the other study, patients were required to take 1,000 mg Korean red ginseng (KRG) 3 times daily. Patients aged 26–70 years with mild or mild to moderate ED were enrolled. The duration of this study spanned a period of 3 months. Sixty patients were recruited, and none dropped out (de Andrade et al., 2007).

### 4.5 *Trigonella foenum-graecum* L. (Fabaceae)

The results of two randomized, double-blind, placebo-controlled clinical trials on *T. foenum-graecum* found that, compared with the baseline and the placebo group, Testofen, the active extract of *T. foenum-graecum*, significantly improved Derogatis Interview for Sexual Functioning-Self Report (DISF-SR) (*p* < 0.01) and AMS scores (*p* < 0.001, *p* < 0.013, respectively) in healthy males (Steels et al., 2011; Rao et al., 2016). These two studies also found that Testofen significantly improved the scores of the sexual arousal domain and orgasm domain, especially the ability to obtain full erections, and the ability, intensity and duration of orgasm (Steels et al., 2011; Rao et al., 2016). One study also found that the average erection time per week (*p* = 0.001) and sexual activity per month (*p* = 0.004) increased significantly after receiving Testofen (Rao et al., 2016).

In one study, the Testofen tablet was supplied by Gencor Pacific Ltd. One tablet contained 300 mg Testofen, as well as 17 mg magnesium, 15 mg elemental zinc and 5 mg pyridoxine. Study subjects were required to take 2 tablets per day. Sixty healthy males aged 25–52 years were recruited, and 6 dropped out. The duration of this study spanned 6 weeks (Steels et al., 2011).

In the other study, 43–70-year-old healthy males were required to take 600 mg Testofen (extracted from *T. foenum-graecum* seed) per day. The duration of this study spanned a period of 3 months. A total of 120 males were recruited, and 9 dropped out (Rao et al., 2016).

### 4.6 *Pistacia vera* L. (Anacardiaceae)

A prospective study on *P. vera* found that *P. vera* significantly improved the IIEF-15 and IIEF-5 scores, as well as the mean peak systolic velocity (*p* = 0.001, *p* < 0.05, *p* = 0.018, respectively). Additionally, the study also found that the levels of total cholesterol (TC) and low density lipoprotein (LDL) in the experimental group decreased, while the levels of high density lipoprotein (HDL) increased (*p* = 0.008, 0.007 and 0.001, respectively), suggesting potential hypolipidaemic effects (Aldemir et al., 2011). In this study, 38–59-year-old patients with ED for at least 12 months were enrolled, and they were required to eat 100 g pistachio (*P. vera*) nuts at lunch for 3 weeks.

The prospective study had only 17 study subjects, and no placebo group was set.

### 4.7 *Withania somnifera* (L.) Dunal (Solanaceae)

*W. somnifera* did not show therapeutic effect on psychogenic ED in a clinical study. In this study, patients with psychogenic ED who had a serum testosterone level of no less than 240 ng/dL were enrolled (Mamidi and Thakar, 2011).

In this randomized, single-blind, placebo-controlled study, patients were required to take 4 *W. somnifera* tablets (dried root powder of *W. somnifera*, 500 mg each) 3 times per day. The duration of this study spanned a period of 2 months. Ninety-five patients were recruited, and 9 dropped out. More than half of the patients had high severity of ED, and more than 80% of the patients had interpersonal conflicts with their partners (Mamidi and Thakar, 2011).

## 5 Mixed botanical medicine

The mixed botanical medicine was either a mixture of herbal plant extracts (Iacono et al., 2012; Sansalone et al., 2014; Quarto et al., 2017; Park et al., 2019; Mirone et al., 2021) or a mixture of active herbal plant ingredients (Shirai et al., 2021). The results of the six mixed botanical drugs showed therapeutic effects on ED.

Among them, a randomized, multicenter, double-blind, placebo-controlled study found that “a new nutritional supplement” [500 mg of *P. ginseng*, 200 mg of *Moringa oleifera* Lam. (Moringaceae), and 50 mg of rutin] combined with tadalafil increased cGMP levels (*p* < 0.05). Patients were required to take the “new nutritional supplement” with/without 5 mg tadalafil once a day. The duration of this study spanned a period of 3 months. Patients aged 38–69 years with mild to moderate ED and with hypertension and/or type 2 diabetes were enrolled. Eighty-six patients were recruited, and 8 dropped out (Mirone et al., 2021).

A prospective, randomized, single-blind, placebo-controlled study found that Tradamix TX1000 (300 mg of alga *Ecklonia bicyclis*, 450 mg of *T. terrestris*, and 250 mg of glucosamine oligosaccharide) improved peak systolic velocity (PSV) and ED symptoms in patients with moderate arterial dysfunction. Patients were required to take Tradamix TX1000 tablets twice a day. The duration of this study spanned a period of 3 months. Two hundred patients were recruited, and 23 dropped out (Sansalone et al., 2014).

A randomized, double-blind study found that compared with tadalafil, Tradamixina (150 mg of alga *E. bicyclis*, 396 mg of *T. terrestris*, and 144 mg of D-glucosamine and N-acetyl-D-glucosamine), with the same main ingredients as Tradamix TX1000, showed a stronger effect of improving ED symptoms, especially in the ED and libido domains of the IIEF-5 scores. In addition, Tradamixina also showed the ability to increase serum testosterone levels. Patients were required to take Tradamixina twice a day. The duration of this study spanned a period of 2 months. Over 60 years of age, patients with reduced libido, with or without ED, were enrolled. Seventy patients were recruited (Iacono et al., 2012).

A randomized, double-blind, placebo-controlled study found that KBMSI-2 [260.53 μg/g Rb1, 543.91 μg/g Rb2, 424.92 μg/g Rc, 377.32 μg/g Re, 1,160.55 μg/g Rf, 703.97 μg/g Rg1, 60.73 μg/g curcumin, 98.66 μg/g allantoin, and 744.13 μg/g loganin, extracted from Ginseng Radix Rubra, *Dioscorea tenuipes* Franch. & Sav. (Dioscoreaceae), *Cornus officinalis* Siebold & Zucc. (Cornaceae), *Lycium chinense* Mill (Solanaceae), and *Curcuma longa* L. (Zingiberaceae)] showed statistically significant improvement in the IIEF-EF domain, intercourse satisfaction domain, and IIEF total scores. Forty-to eighty-year-old patients with ED were enrolled and required to take 6 g of KBMSI-2 twice per day. The duration of this study spanned a period of 2 months. Forty-four patients were recruited, and 18 dropped out (Park et al., 2019).

IDIProst^®^ Gold [100 mg of *C. sativus* stigma dry extract 0.3% minimum safranal, 120 mg of *Pinus massoniana* Lamb. (Pinaceae) bark extract 95% minimum proanthocyanidins, and 320 mg of *Serenoa repens* (W. Bartram) Small (Arecaceae) fruit oil extract 88% minimum fatty acids] significantly improved IIEF-5, IPSS, and QoL (question of life) scores, especially in the 40–60 age group. In addition, IDIProst^®^ Gold increased Qmax (maximum urinary flow rate) variation in subjects under 50 years old but had no effect on subjects over 50 years old. The detailed study design was not mentioned. Patients aged 20–75 years with concomitant lower urinary tract symptoms (LUTS) and ED were enrolled and required to take one IDIProst^®^ Gold capsule per day. The duration of this study spanned a period of 3 months. A total of 164 patients were recruited, and 24 dropped out (Quarto et al., 2017).

A randomized, double-blind, placebo-controlled, crossover study found that “supplement drink” (testofen 600 mg/d extracted from *T. foenum-graecum*, L-citrulline 800 mg/d, resveratrol 300 mg/d, and caffeine 40 mg/d) showed significantly improved the IIEF total scores, especially in the domains of IIEF-1, IIEF-2, IIEF-7, IIEF-9, IIEF-10, IIEF-13, and IIEF-15. The duration of this study spanned a period of only 5 weeks (2 weeks for “supplement drink” and 2 weeks for placebo, with 1 week for wash-out). Thirty-to forty-year-old patients with ED were enrolled. Twenty patients were recruited, and none dropped out. In addition, the study lacked information on comorbidities that could affect sexual function, such as diabetes (Shirai et al., 2021).

## 6 Adverse events of botanical drugs

There were 24 studies that analysed the incidence of adverse events (Tables 1–3). Overall, no serious adverse events were reported in clinical studies. Among these 24 studies, 11 did not report any adverse events. The reported adverse events included gastrointestinal symptoms, headache, facial flushing, dizziness, and insomnia, and these symptoms were mostly mild to moderate. Among all the adverse events, headache and gastrointestinal symptoms were most common. One clinical trial analysed the incidence rate of adverse events between the combined use of “a new nutritional composition” (500 mg *P. ginseng*, 200 mg *M. oleifera*, and 50 mg rutin) and tadalafil and the use of tadalafil alone. No difference in the incidence rate of adverse events between the two groups was found (Mirone et al., 2021). This clinical trial also reported that one study subject experienced insomnia in the combined use of the two drugs group, while no study subject experienced insomnia in the use of tadalafil alone group (Mirone et al., 2021). A clinical trial of *G. biloba* reported that in addition to headache and gastrointestinal symptoms, the experimental subjects who took *G. biloba* also experienced sedation and increased oral intake (Kang et al., 2002). Another clinical trial of *G. biloba* reported that the experimental subjects experienced paraesthesia fingers, but the incidence rate was not statistically significant compared to the placebo group (Wheatley, 2004). Similarly, a clinical trial of *C. sativus* reported that the experimental subjects experienced nausea, daytime drowsiness, decreased appetite, dry mouth, nervousness, restlessness, morning drowsiness, and increased appetite. Although this clinical trial had more different adverse events compared to other studies, statistical analysis found that the incidence rate of these side effects was not significantly different from the placebo group and mostly at the mild to moderate degree and therefore tolerable (Modabbernia et al., 2012). In a clinical trial of *E. longifolia*, two severe adverse events, low back pain and liposome, were reported in the experimental group, but they were not considered to be associated with the intervention measure. In addition, this clinical trial also recorded some mild to moderate adverse events, such as upper respiratory tract infections, generalized body ache, conjunctivitis, infected chalazion, ankle pain, achilles tendinitis, herpes zoster, and R index finger pain, but these adverse events were not considered to be associated with the intervention measure (Ismail et al., 2012).

**TABLE 3:** Mixed botanical drugs.

Name	Botanical ingredients	Type of study	Study subject	Intervention measure	Therapeutic effects	Adverse events
Tradamixina	1) Alga *Ecklonia bicyclis*	Randomized double-blind	>60 years old patients with reduced libido, with or without ED	“Tradamixina” twice per day for 2 months (150 mg of alga *E. bicyclis*, 396 mg of *T. Terrestris* and 144 mg of D-Glucosamine and N-Acetyl-D-Glucosamine)	Erectile function, libido, total and free testosterone levels improved in elderly men, without side effects of tadalafil. Kang et al. (2002)	None
	2) *Tribulus terrestris* (PDE5-i)					
Tradamix TX1000	1) Alga *E. bicyclis*	Randomized single-blind placebo	Patients with mild to moderate ED	One tablet orally twice a day for 3 months (containing 300 mg of alga *E. bicyclis*, 450 mg of *T. terrestris* and 250 mg of glucosamine oligosaccharide)	PSV, MSHQ-EjD, SQoL-M, IIEF-EF, intercourse satisfaction, orgasmic function, sexual desire, overall satisfaction domains improved. Wheatley, (2004)	None
	2) *T. terrestris* (PDE5-i)					
KBMSI-2	1) *Dioscorea tenuipes*	Randomized double-blind placebo parallel	40∼80 years old patients with ED	6 g of KBMSI-2 (260.53 μg/g Rb1, 543.91 μg/g Rb2, 424.92 μg/g Rc, 377.32 μg/g Re, 1,160.55 μg/g Rf, and 703.97 μg/g Rg1, 60.73 μg/g curcumin, 98.66 μg/g allantoin, and 744.13 μg/g loganin) twice per day for 8 weeks, at least 1 h after food intake	IIEF-EF domain and intercourse satisfaction domain improved. Stein et al. (2018)	Only one has a mild itching sensation during the study and in the follow-up period
	2) *Cornus officinalis*					
	3) *Lycium chinense* Mill					
	4) *Curcuma longa* Linn					
IDIProst^®^ Gold	1) *Serenoa repens* (PDE5-i)	Not mentioned	20∼75 years patients with concomitant LUTS and ED	One capsule per day for 3 months (containing 100 mg of *C. sativus* stigma dry extract 0.3% minimum safranal, 120 mg of *P. massoniana* bark extract, 95% minimum proanthocyanidins and 320 mg of *S. repens* fruit oil extract 88% minimum fatty acids)	IIEF-5 and QoL improved greater in the 40–60 age group than in all the age groups The score of Qmax variation improved under the age of 50, while not improved over 50. Shin et al. (2023)	None
	2) *Crocus sativus*					
	3) *Pinus massoniana*					
“A new nutritional supplement”	1) *Panax ginseng*	Multicenter randomized double-blind placebo	38∼69 years patients with mild to moderate ED, and with hypertension and/or type 2 diabet	Tadalafil 5 mg once daily plus nutritional supplement contained *P. ginseng* (500 mg), *M. oleifera* (200 mg) and rutin (50 mg) once daily for 3 months	IIEF-5 score and cGMP levels improved. Zenico et al. (2009)	1) Headache
	2) *Moringa Oleifera* (PDE5-i)					2) Nasal congestion
	3) Rutin					3) Back pain
						4) Myalgia
						5) Insomnia
						6) Dizziness
						7) Flushing
						(The rate of adverse events is comparable between the two groups. *p* = 0.38)
“Supplement drink”	1) Testofen (extracted from *Trigonella foenum-graecum*)	Randomized double-blind placebo cross-over	30∼47 years old patients with ED	The active drink (testofen 600 mg/d, L-citrulline 800 mg/d, resveratrol 300 mg/d, and caffeine 40 mg/d) for 14 days, then a 7-day washout period	IIEF total score improved, especially in the domains of IIEF-1, -2, -7, -9, -10, −13, and −15. Gonzales et al. (2002)	None
	2) Resveratrol					

PDE5-i, phosphodiesterase-5, inhibitor; ED, erectile dysfunction; PSV, peak systolic velocity; MSHQ-EjD, Male Sexual Health Questionnaire-Ejaculation Disorder; SQoL-M, sexual quality of life instrument for men; IIEF-EF, International Index of Erectile Function-Erectile Function; LUTS, lower urinary tract symptoms; QoL, question of life; Qmax, maximum urinary flow rate; cGMP, cyclic guanosine monophosphate.

## 7 Perspective of future studies and clinical applications

Although most clinical studies for the included botanical drugs are randomized double-blind placebo-controlled trials, some limitations should be noted. The age and comorbidities of participants vary in different studies. For example, some studies enrolled patients over 40 years old, while one study enrolled patients approximately 18–45 years old. Some studies have enrolled androgen deficiency of aging males. Interestingly, the intervention method for one study was to rub the *C. sativus* gel. In addition, the sample size of most studies is limited. The above factors limit the extension of the conclusion. Clinical trials enrolling more representative populations with larger sample sizes are needed in the future to confirm the efficacy of botanical drugs in treating ED. Encouragingly, most clinical trials indeed demonstrate the efficacy of botanical drugs in treating ED without significant adverse effects. These botanical drugs may be used along with PDE5-i or alone to treat ED in clinical practice.

## 8 Conclusion

Overall, this review summarizes several botanical drugs with promising therapeutic effects for ED in clinical studies without significant adverse effects. Further placebo-controlled or head-to-head randomized controlled trials are needed to verify these therapeutic effects, and determine the appropriate usage and dosage and target patients. In addition, further pharmacology experiments need to be conducted for the screening of active compounds and further drug development.
